# Correction: Composing Problem Solvers for Simulation Experimentation: A Case Study on Steady State Estimation

**DOI:** 10.1371/journal.pone.0099147

**Published:** 2014-05-27

**Authors:** The PLOS ONE Staff

## Notice of Republication

This article was republished on April 30, 2014, correct the figure order. Please download this article again to view the correct version. The originally published, uncorrected article and the republished, corrected article are provided here for reference.

## Supporting Information

File S1 Originally published, uncorrected article.
(PDF)

File S2 Republished, corrected article.
(PDF)
