# Supplementary material for: Composing Problem Solvers for Simulation Experimentation: A Case Study on Steady State Estimation
Source: PLoS One. 2014 Apr 4;9(4):e91948. doi: 10.1371/journal.pone.0091948 (PMC3976265; doi:10.1371/journal.pone.0091948)
Supplement: File S1 — Supporting figures and tables. Text S1. A Problem Solver Interface. Figure S1. Sequence diagram depicting the iterated communication between answer function and a problem solver. Figure S2. Example scheme for managing the communication between a simulator producing time series for a steady state estimator. Text S2. Base-Line Steady State Estimator Results on the Training Data. Figure S3. Mean deviation and success rate of the tested steady state estimators applied on problem data. Figure S4. Mean deviation and success rates of the tested steady state estimators applied on time series with different amounts of noise. Table S1. Steady State Estimator Evaluation Results on Simulation Data. (PDF) [file pone.0091948.s001.pdf]

Text S1 - A Problem Solver Interface

We use a general interface for iterative problem solvers, also called *solvers* in the following. It allows a unified handling of algorithms that can be composed as described in the next section. The interface comprises two basic methods: the method *init* is responsible for initializing the problem solver, i.e., setting it up for the problem solving process. The method *solve* realizes the problem solving process itself. It is detailed in the following.

As explained in the introduction, problem solving in simulation experiments usually follows a general scheme that is based on a simple input/output behavior. An example for this is a steady state estimator, which estimates a steady state statistic (e.g., mean) of a given time series. The input is the information obtained about the problem so far, e.g., the available data points of a time series that have been generated by executing simulation steps. The output is the result produced by the problem solver when applied on the received input, e.g., the estimated steady state statistic produced by a steady state estimator.

This input/output behavior is mirrored in the *solve* function's signature, which, given a problem space $\mathbb{P}$ (e.g., the set of possible time series) and a set of possible results RES (e.g., the possible estimated steady states), could be described by:

$$solve : \mathbb{P} \rightarrow RES \tag{1}$$

This signature, however, does not incorporate iterated problem solving that is necessary in cases where not all required information about the problem might be initially available. In these cases, problem solvers need the ability to request further information to handle the problem in additional iterations[1]. For instance, in steady state estimation, the time series is generated iteratively by a simulation algorithm. If the length of the time series is not sufficient for a confident estimation, more time points have to be generated to produce additional data for the estimator, and the estimation has to be repeated.

We use a request-answer scheme, for enabling problem solvers to request for additional data. A problem solver can formulate a request from the set of possible requests REQ (in the steady state estimation case, requests for additional time points of the time series).

To consider problem iterations, instead of static problems, a problem solver may need a state for storing (partial) results of previous iterations, e.g., many steady state estimators keep track of previous results to avoid recalculations. The state of the previous iteration can be considered as input, and the resulting state of the current iteration as output. Consequently, the state includes all information that have to be available for the problem solver: *algorithm state information (AS)* comprising all relevant information for the next iteration, *result information (RES)* representing the solution of the current iteration, and *request information (REQ)* representing additional data required for the next iteration. The set of *problem solver states* $\mathbb{S}$ can, thus, be defined as:

$$\begin{aligned}\mathbb{S} = \{(as, res, req) | as \in AS,\\ res \in RES, req \in REQ\},\end{aligned} \tag{2}$$

All in all, given a set of problem solver states $\mathbb{S}$, the *solve* function of an iterated problem solver PS has the following signature:

$$solve : \mathbb{P}_{it} \times \mathbb{S} \rightarrow \mathbb{S} \tag{3}$$

Iterated problems from $\mathbb{P}_{it}$ include the initial problem description, and a *request-answer history* $H \in \mathbb{H}$. The history is a sequence $H = h_1, \ldots, h_n$ with $h_i \in (REQ, ANS)$, i.e., it holds issued requests from REQ and corresponding answers from the set of possible answers ANS comprising additional information about the problem. The set of iterated problems $\mathbb{P}_{it}$ is defined by:

$$\mathbb{P}_{it} = \mathbb{P} \times \mathbb{H}, \tag{4}$$

[1]Problem solvers that do not work iteratively can be treated as iterative problem solvers needing only one iteration.

To generate answers for requests, an *answer function* η is applied:

$$\eta : REQ \rightarrow ANS \tag{5}$$

The answer function is not part of the problem solver itself, but rather a part of the problem description and consequently depends on the kind of problem to be solved. For instance, the data points of the problem time series of a steady state estimation are usually generated by simulation, making the simulation engine the answer function.

Managing the problem solving process To solve a problem effectively, requests as well as answers have to be integrated in the problem definition in each iteration. To make that happen, a management component (e.g., a predefined workflow) is necessary that interacts with the interface of the problem solver PS and the answer function η. Figure S1 shows a sequence diagram for all three components when solving

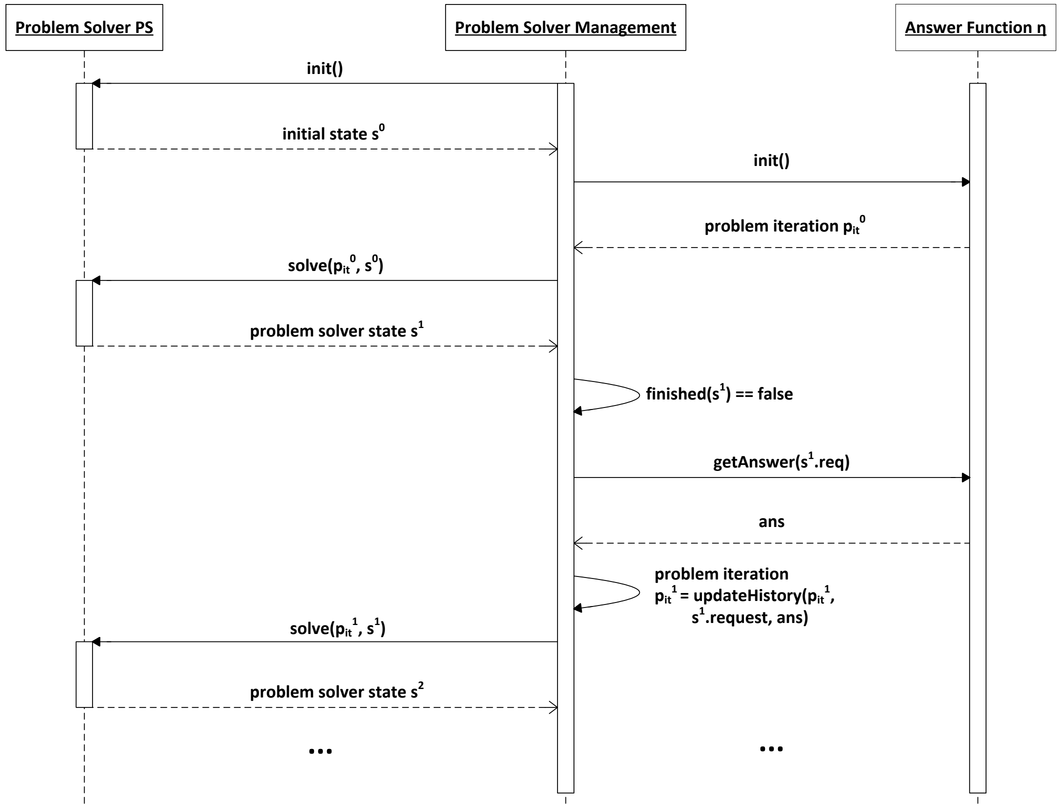

Figure S1. Sequence diagram depicting the iterated communication between answer function (Equation 5), e.g., representing simulator, and a problem solver, e.g., steady state estimator. The communication is controlled by a managing component that is also responsible for updating the problem description $p \in \mathbb{P}_{it}$.

a problem $p \in \mathbb{P}_{it}$, iteratively. During the initialization of the problem solver PS, an initial state $s_0 \in \mathbb{S}$ is generated. Similarly, the representation of the first problem iteration, $p_{it}^0 \in \mathbb{P}_{it}$, only includes the problem itself and an empty request history; it is created by initializing the answer function η. Figure S2 depicts a

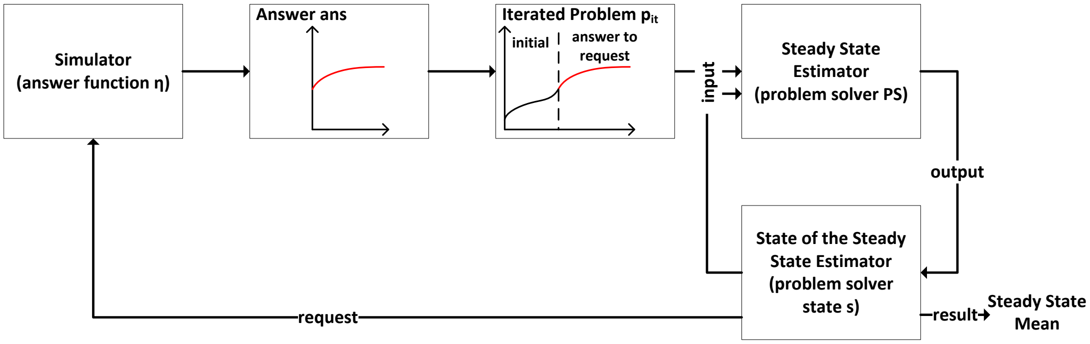

Figure S2. Example scheme for managing the communication between a simulator producing time series for a steady state estimator. The steady state estimator works on its previous state and a time series, generated by the simulator, as input. After each estimation iteration, the estimator returns its state, comprising a request for the simulator, which executes the next simulation steps. Thereby, a new segment of the time series is generated and added to the problem time series, which is used in the next estimation iteration.

steady state estimation example, where the initial problem is a time series. With both elements, problem solver state s_0 and problem iteration p_0, the `solve` method (Equation 3) of the problem solver is called. It returns a successor state holding the request for getting additional information about the problem, e.g., in steady state estimation, this would be a boolean value denoting that additional data points of the time series are required. Afterwards, the answer function η (Equation 5) is called by the management component. The resulting answer (an additional segment of the time series in the steady state estimation example) in combination with the request is added to the request history of the problem, leading to a new problem iteration, which now includes the initial problem description and a request history comprising the first request-answer pair. After each iteration, the management component calls the method `finished` to check whether a final problem solver state is reached. This function highly depends on the problem at hand, e.g., if the problem is a time series where the steady state shall be estimated, `finished` would return `true`, as soon as an estimate can be given, whereas in optimization the problem solving is usually finished when given cancel criteria are met. The whole process is repeated until `finished` returns `true`.

Text S2 - Base-Line Steady State Estimator Results on the Training Data

Figure S3 shows the performance results of the investigated steady state estimators applied on the problem data described in Section 5.1.5. Performance measures include the mean deviations between estimated and real steady state, as well as the success rates, i.e., the ratio between successful detections of the time series warm-up phases and overall estimator applications. It is visible that the used steady state estimators behave differently. The goodness of fit steady state estimator has by far the best deviation of less than 0.002, but also the worst success rate with 0.5. The good accuracy of this estimator might result from the rare cases where it finds the warm-up phase's end (which might be more easy to handle). This explanation is supported by the fact that the cases where this estimator is unsuccessful are all false negatives.

The stop crossing and batch means estimators seem to have an opposite behavior. Both produce false positives in cases where they are unsuccessful in finding the end of the warm-up phase. However, the

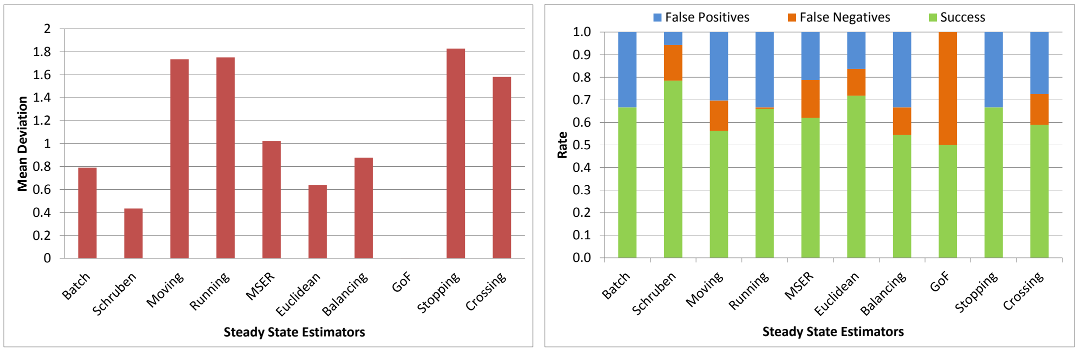

Figure S3. Mean deviation and success rate of the tested steady state estimators applied on problem data.

success rate of more than 0.5 indicates that they do not always find an end of the warm-up phase and that they are very reliable in these cases. This could be an important feature to consider for composition.

Schruben's steady state estimator has the best success rate (0.78) of all tested estimators and the second best deviation (0.43) The remaining steady state estimators represent different trade-offs between success rate and deviation.

Altogether, the ability of detecting the end of the warm-up phase seems to have an impact on the quality of steady state mean estimates.

Furthermore, different features of time series influence the performance of steady state estimators, as the following examination of estimators performance on trajectories with different induced noises illustrates. Figure S4 shows the results of the steady state estimators on time series with 0 and 10 percent noise. For the MSER steady state mean estimator, the fact whether noise exists at all, has a high impact on the estimation results. This is visible at the mean deviation (between estimated and real steady state) being much higher on time series without noise than with noise (independently from the concrete amount of noise). Furthermore, if noise exists, the MSER estimator has a much better success rate and does not produce false any negatives. With respect to success rates, this estimator is the most reliable of the tested estimators, when working on time series that comprise noise. Only the euclidean distance estimator is slightly better (< 3 percent) on time series with a noise of 5 percent, however it performs worse as the noise is increased. Similar to the MSER estimator, the crossing mean estimator performs better with noise than without noise, as its success rate rises from 0.33 without noise to above 0.65 if noise exists in the time series.

In contrast to MSER and crossing mean estimator, the goodness of fit estimator performs better without noise than with noise. It has a success rate of 1.0 on time series without noise and does not find any steady state in time series where noise exists (unsuccessful cases are always false negatives).

The performance of Schruben's estimator depends on the noise, as its mean deviation between estimated steady state mean and real steady state rises with the noise level in the time series it is applied to.

All in all, the amount of noise seems to be an interesting feature for finding the most suitable steady state estimator. For instance, if no noise can be found it might be reasonable to use the goodness of fit estimator to find the end of the warm-up phase. With low amounts of noise (around 5 percent) the euclidean distance estimator should be used, while MSER could be a good choice with higher amounts of noise. However, noise needs to be measured properly to make such decisions. This can be challenging, if the noise cannot be derived from the time series generator, which is the case in most real-world scenarios.

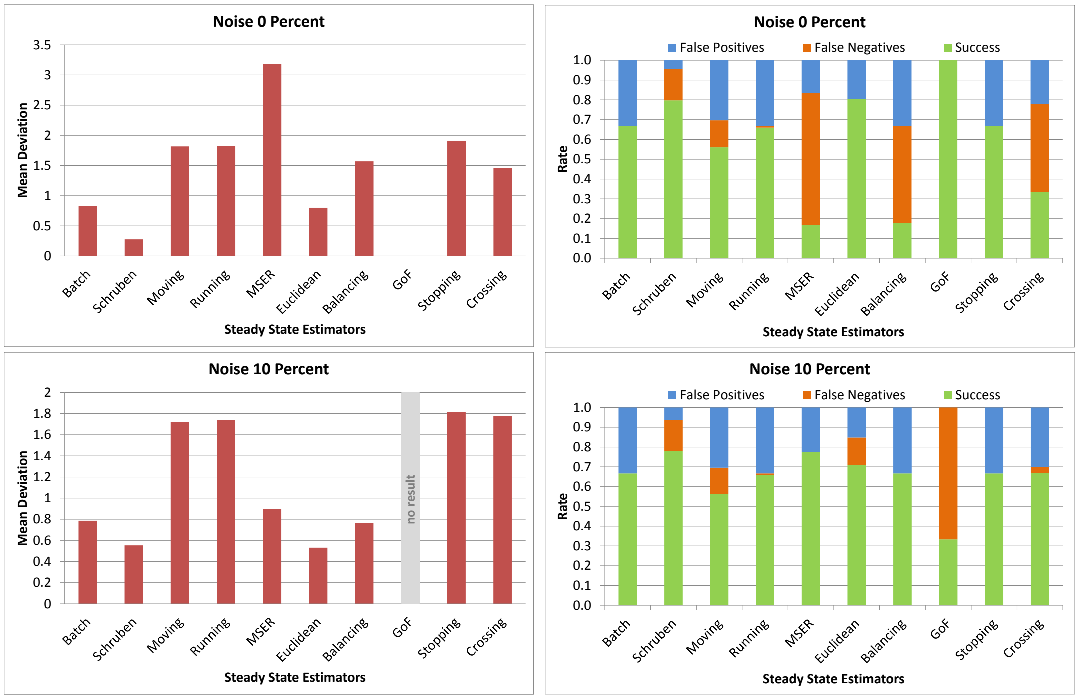

Figure S4. Mean deviation and success rates of the tested steady state estimators applied on time series with different amounts of noise.

Table S1 - Steady State Estimator Evaluation Results on Simulation Data

	MgCl2		NaCl		kNa2Cl	
Steady State Estimator	Deviation	Chunks	Deviation	Chunks	Deviation	Chunks
Batch Means	0.16678	-2.23	0.04820	1.25	0.05450	-0.91
Schruben's Test	0.04095	1.11	0.03342	2.74	0.04798	-0.75
Moving Windows	0.00434	3.48	0.01112	3.93	0.01301	3.35
Running Mean	NaN	10	0.05253	1.85	0.06355	2.94
MSER	0.30774	-3.82	0.06069	0.68	0.08334	-1.72
Euclidean Distance	0.46379	6.7	NaN	10	NaN	10
Balancing Mean	0.23564	-3	0.03673	1.92	0.06023	-0.97
Goodness of Fit	0.13081	1.3	NaN	10	0.09618	5.88
Stop Crossing Mean	0.07449	9.96	NaN	10	NaN	10
Crossing Mean	NaN	10	NaN	10	NaN	10
Synthetic	0.00280	2.62	0.00711	2.43	0.01014	1.55

	HCl		Mapk		TCR	
Steady State Estimator	Deviation	Chunks	Deviation	Chunks	Deviation	Chunks
Batch Means	0.00006	0.03	0.04565	-2	0.27655	3
Schruben's Test	0.00382	0.99	0.05388	-2.03	0.94547	-1
Moving Windows	0.00232	3	0.03451	-1.28	NaN	10
Running Mean	0.00447	-0.05	NaN	10	NaN	10
MSER	0.00025	0.07	0.04711	-1.7	0.94547	-1
Euclidean Distance	NaN	10	NaN	10	NaN	10
Balancing Mean	0.00206	0.15	0.08645	-2.18	NaN	10
Goodness of Fit	NaN	10	0.31742	9.74	NaN	10
Stop Crossing Mean	NaN	10	NaN	10	NaN	10
Crossing Mean	NaN	10	NaN	10	NaN	10
Synthetic	0.00099	0.29	0.01489	0.82	0.05453	5
