# Supplementary material for: Correction: Composing Problem Solvers for Simulation Experimentation: A Case Study on Steady State Estimation
Source: PLoS One. 2014 May 27;9(5):e99147. doi: 10.1371/journal.pone.0099147 (PMC4035329; doi:10.1371/journal.pone.0099147)
Supplement: File S2 — Republished, corrected article. (PDF) [file pone.0099147.s002.pdf]

OPEN ACCESS Freely available online

PLOS ONE

Composing Problem Solvers for Simulation Experimentation: A Case Study on Steady State Estimation

Stefan Leye*, Roland Ewald, Adelinde M. Uhrmacher

Institute of Computer Science, University of Rostock, Rostock, Germany

Abstract

Simulation experiments involve various sub-tasks, e.g., parameter optimization, simulation execution, or output data analysis. Many algorithms can be applied to such tasks, but their performance depends on the given problem. Steady state estimation in systems biology is a typical example for this: several estimators have been proposed, each with its own (dis-)advantages. Experimenters, therefore, must choose from the available options, even though they may not be aware of the consequences. To support those users, we propose a general scheme to aggregate such algorithms to so-called synthetic problem solvers, which exploit algorithm differences to improve overall performance. Our approach subsumes various aggregation mechanisms, supports automatic configuration from training data (e.g., via ensemble learning or portfolio selection), and extends the plugin system of the open source modeling and simulation framework James II. We show the benefits of our approach by applying it to steady state estimation for cell-biological models.

Citation: Leye S, Ewald R, Uhrmacher AM (2014) Composing Problem Solvers for Simulation Experimentation: A Case Study on Steady State Estimation. PLoS ONE 9(4): e91948. doi:10.1371/journal.pone.0091948

Editor: Frederic Amblard, University Toulouse 1 Capitole, France

Received August 30, 2013; **Accepted** February 18, 2014; **Published** April 4, 2014

Copyright: © 2014 Leye et al. This is an open-access article distributed under the terms of the Creative Commons Attribution License, which permits unrestricted use, distribution, and reproduction in any medium, provided the original author and source are credited.

Funding: The research was funded by the German Reseach Foundation (DFG), under the projects Alesia and CoSA. The funders had no role in study design, data collection and analysis, decision to publish, or preparation of the manuscript.

Competing Interests: The authors have declared that no competing interests exist.

* E-mail: stefan.leye@uni-rostock.de

Introduction

The identification of steady states is essential for many applications of computational biology, e.g., to study human cancer [1], T-helper cell differentiation [2], T cell receptor (TCR) signaling [3], or cell cycles of yeast types [4]. Several approaches to identify steady states exist. Analytical approaches typically investigate the full state space of a model. While they are suitable for some kinds of models, e.g., boolean networks [5], a complete state-space coverage is often infeasible for more complex models. In such cases, steady states can be estimated via simulation.

Simulation-based *steady state estimation* is independent of the modeling formalism, as it merely assumes that trajectories through a model's state-space can be generated. To understand the steady state behavior of stochastic models, it is often necessary to generate multiple trajectories that cover a spectrum of possible paths through the state-space. For each trajectory, one observes how the quantity of interest, e.g., the amount of a chemical species, changes over time. The resulting time series are then analyzed to estimate a steady state statistic, e.g., the mean. Various methods have been proposed for this task, e.g., [6–11]. Experimenters need to decide which method to use.

However, steady state estimation is only one of many sub-tasks to be carried out. Similar decisions must be made for other tasks, e.g., regarding parameter optimization, simulation, or further data analysis. Due to the variety of challenges, a typical user cannot be expert in all relevant disciplines, and hence will find it difficult to make these decisions. This calls for a better support in conducting simulation experiments. While our previous work focused on automatically selecting simulation algorithms [12], the other sub-tasks of simulation experiments [13] need to be covered as well. Moreover, the selection of an individual algorithm is only one way to solve this problem; it is a special case of the general approach to *compose* several algorithms for a certain task into a single algorithm.

To support this, general-purpose simulation systems need to offer generic composition mechanisms, which should also reflect the specifics of simulation experiments. For example, sub-tasks of simulation experiments are usually executed iteratively. Such mechanisms should also be easy to tune to the given application domain, to improve their performance. This is especially relevant for computational systems biology, e.g., Ghosh et. al. [14] demand that "*software tools and resources for systems biology need to be tailored to their intended applications in order to achieve the objectives of novel biological discoveries*".

In this paper, we present an approach to compose simulation experiment methods that are fine-tuned toward their application domain. One of our main goals is to make simulation-based steady state estimation more accessible to users who lack the experience to select a suitable method. Nevertheless, our approach is generic, i.e., it also enables a composition of other simulation experiment tasks, such as parameter optimization. It is based on the notion of a synthetic problem solver (called SPS in the following and defined in Equation 3) component, which incorporates a set of *base-line* problem solvers, e.g., steady state estimation methods, and combines them to improve the overall performance. The main advantage is that users do not have to manually select the best suited algorithm for each concrete problem. Instead, a domain

expert provides a set of representative example problems, from the same problem domain as the concrete problem to be solved. A synthetic problem solver is then trained on those example problems to learn a suitable algorithm composition for this problem domain. It will thus be able to find a good solution for the concrete problem, without involving the user any further.

We assume that each base-line problem solver can solve problems of the given type alone, and that all solvers for this problem type work on the same input data, e.g., time series in case of steady state estimation. Our approach involves two major steps: a) it evaluates the base-line problem solvers on a set of representative problems, and b) it analyzes the collected data to generate an SPS instance with superior performance, e.g., in terms of robustness. To prototype the creation and usage of synthetic problem solvers, we extend the modeling and simulation framework James II [15,16] to accommodate them as synthetic (i.e., user-specified, automatically generated) plugins. By extending James II 's plugin system, we ensure a high degree of flexibility with respect to both the base-line algorithms and the mechanisms for combining them. To illustrate the generality of our approach, we discuss related approaches for algorithm composition, and later show how they can be realized with the SPS concept. To show the effectiveness of our approach, we describe an SPS for steady state estimation. It is based on ten base-line problem solvers and performs well on simulation output data from seven biochemical models: six simple reaction networks from [17] and a T cell receptor (TCR) signaling model from [3].

Materials and Methods

2.1 Background and Related Work

Ghosh et. al. [14] structure tools and methods applied in systems biology according to iterative cycles of experimentation, data acquisition and analysis, modeling, and computational analysis. They stress the need for standardization and interoperability between tools and demand unifying platforms to make tools accessible in a consistent manner, in order to improve productivity and reduce possible errors. James II is such a platform for modeling, simulation, and experimentation [15,16]. It is the basis of the presented composition mechanism and offers various methods at different points of the experiment process, e.g., simulators, parameter optimization methods, and steady state estimators.

The basic idea of automatically composing software components is anything but new. The challenge we tackle here is to integrate such functionality into simulation software systems, in a way that allows both a high degree of automation and also high flexibility when it comes to the *way* in which the software components are composed. Therefore, we briefly review relevant formal methods to compose algorithms (Section 2.1.1) and then discuss automatic component composition in other software systems (Section 2.1.2).

2.1.1 Composition Schemes. Composing algorithms is typically associated with applying a function to the results of another function (e.g., via function composition: $f \circ g$). Another notion, on which we focus here, is composing algorithms $a_1, \ldots, a_n \in \mathbb{A}$, all of which are applicable to elements from the same problem space $\mathbb{P}$, so that the overall performance of a composite algorithm $c(a_1, \ldots, a_n)$ is superior to that of $a_1, a_2, \ldots,$ and a_n, respectively.

Rice's formalization of the so-called *algorithm selection problem* (ASP) can be regarded as a general scheme to compose better-performing algorithms [18]. In principle, it deals with selecting the most suitable (base-line) algorithm from an algorithm space $\mathbb{A}$ to solve a problem x from problem space $\mathbb{P}$. To achieve this goal, features $f \in \mathbb{F}$ are extracted from x, using an extraction mapping $F : \mathbb{P} \to \mathbb{F}$. A (typically unknown) performance mapping $p : \mathbb{A} \times \mathbb{P} \to \mathbb{R}^k$ defines the (multi-faceted, hence k-dimensional) performance of algorithms (in $\mathbb{A}$) on problems (in $\mathbb{P}$). Additionally, a norm $\|\cdot\|$ that depends on user criteria $w \in \mathbb{R}^k$ is introduced to characterize algorithm performance with a single value (for algorithm comparison). User criteria may, for example, define the desired balance between the accuracy and the speed of an algorithm. The problem is now to find a selection mapping $S : \mathbb{F} \times \mathbb{R}^k \to \mathbb{A}$, i.e., a function that takes the features $F(x) \in \mathbb{F}$ of a problem $x \in \mathbb{P}$ and the user criteria $w \in \mathbb{R}^k$ to select the best-performing algorithm $a \in \mathbb{A}$. Rice considers this to be an approximation problem, i.e., a selection mapping S can be found with techniques from machine learning. The approach allows, for example, to support users in selecting a suitable execution algorithm for a model [19]. A fully automatic selection is also feasible, e.g., it could considerably speed up the simulation of chemical reaction networks in James II [12]. However, most implementations of this approach are focused on recommender systems for scientific software in general, i.e., algorithms are selected but not composed (e.g., see [20]).

Instead of selecting a single base-line algorithm, the fundamental idea of *algorithm portfolios* is to apply a set $A \subseteq \mathbb{A}$ of algorithms to the same problem and to combine their results afterwards. This approach originated in finance, where financial assets are bundled into portfolios that maximize the expected return, given the risk aversion preferences of an investor [21]. Instead of a selection function, portfolios assign weights to each available algorithm, and use them to combine the algorithms' results afterwards. Portfolio approaches have been successfully applied to SAT solving (e.g., [22]) and other NP-hard problems (e.g., [23,24]), as well as the simulation of chemical reaction networks in James II [12]. Portfolio-based approaches receive attention in many other domains, e.g., for valuating and managing biodiversity [25], and for exploring biological robustness and a suitable balance of fragility and performance in cell biological systems [26].

Similar to algorithm portfolios, algorithm ensembles typically apply multiple algorithms to a single problem. In contrast to portfolios, however, the application of algorithms is not defined by weights. Instead, an additional function is learned that considers the results of the algorithms in $\mathbb{A}$ to generate a result. Algorithm ensembles are predominantly used in machine learning, where various methods exist to train the base-line algorithms on (subsets of) input data first, and to then train a function that operates on their results, afterwards. The learned function could, for example, decide which base-line algorithm a_i performs well in which region of the problem space $\mathbb{P}$, i.e., which result to choose in which situation [27,28]. In machine learning, the latter approach is called meta-learning; it can be tackled with methods to solve the ASP [29]. This approach of algorithm composition from empirical data is promising, since the results of the base-line algorithms in $\mathbb{A}$ may reveal important properties of the problem at hand. Ensemble methods are widely used in bioinformatics, e.g., for proteomics data analysis [28].

All composition schemes discussed above imply some form of learning: either a selection function, portfolio weights, or an ensemble function shall be learned, typically from past performance data. Learning these elements can also be done online, i.e., during a sequence $x_1, \ldots, x_n \in \mathbb{P}$ of problems being solved. An example for such an approach is the adaptive online time allocation (AOTA) method [30]. It adapts the weights of algorithms according to their performance observed so far, by

maintaining a history of problem features and performance measurements, which are also recorded during the solution of a single problem. Based on this history, it is possible to predict the additional computing time an algorithm a_i needs until it solves a problem x_j, and to allocate resources accordingly. This approach allows to learn algorithm portfolios incrementally [31] and lends itself to an integration of various learning methods, e.g., reinforcement learning [32]. Reinforcement learning has already been used to speed up the simulation of chemical reaction networks [33].

2.1.2 Automated Composition in Software Systems. In practice, an automated composition of software components is particularly relevant for (self-)adaptive software, i.e., software that is designed to adapt to changes in the user's needs or changes in its environment. To achieve this, adaptive software is flexible with respect to the methods for handling a problem, i.e., it does not rely on one design decision for an algorithm but employs different ones, continuously re-deciding which one to use in the given context. Norvig and Cohn [34] list five key technologies that form the foundation of adaptive software: dynamic programming languages, agent technology, decision theory, reinforcement learning, and probabilistic networks. While we use a programming language that is mostly static (Java) and do not rely on the agent metaphor, the composition scheme we put forward in Section "Synthetic Problem Solvers" can integrate elements that rely on the last three technologies. Each of those could be used within a composed algorithm $c(...)$ to reason about the performance of the $a_1,\ldots,a_n \in \mathbb{A}$ (see Section 2.1.1) and adapt itself accordingly.

In [35], McKinley et al. present a taxonomy of composition techniques for adaptive software. From their point of view, we realize a dynamic composition of algorithms at runtime. From the perspective of autonomous computing [36], our approach allows to dynamically re-configure simulation systems by (re-)creating composed algorithms that are better tuned to the tasks at hand. Via learning, the simulation system gets aware of its context, i.e., the problems it shall be applied to, and can optimize itself.

While we strive to automate the overall process of composing an algorithm $c(\ldots)$, it should still be triggered manually by a user. This is a major difference to the autonomous computing setting, but it is necessary: a user needs to define the performance metric of interest and the problems to be used for training. Although the need for manual intervention could be perceived as a drawback, it also helps a practitioner to be aware of the available algorithmic alternatives and the underlying assumptions of their composition, e.g., that the training set of problems is representative for the tasks to come.

Automatic composition has also been addressed in other fields, but mostly for enabling the execution of new tasks that require interaction between the components (e.g., web services). Such a composition can be supported via languages or language features, such as language-integrated queries (LINQ, e.g., see [37]), or by additional tools. For example, K-BACEE [38] allows to automatically evaluate component 'ensembles'. These methods are fundamentally different from our approach: we aim to compose complete algorithms, each *already* designed to fulfill the task at hand. Our main motivation for this is to improve performance metrics, such as the quality of the results.

Ostertag et al. [39] propose AIRS, an AI based library for software reuse, which is designed to browse software libraries for (reusing) components and packages that best meet given requirements. As components are described according to features, which represent classification criteria, they can then be selected according to the similarity between description and target requirements. While the application domain of AIRS (browsing of components) differs from our approach (orchestration of algorithms to gain improved results), they share one key idea, namely to represent components (i.e., algorithms) by their features to assess their suitability for the problem at hand. In the domain of simulation, adaptation is typically not realized as a property of the simulation system, but as a feature of specific algorithms. For example, there are many approaches for adaptive algorithms in the field of parallel and distributed discrete-event simulation (e.g., [40–45]). Similarly, some numerical integration methods in continuous simulation realize error control by adaptation, e.g., by changing step-size or using a different order method [46].

2.2 Synthetic Problem Solvers

We propose a general structure for an automated composition of algorithms, which is called *synthetic problem solver (SPS)*. The SPS contains the logic to orchestrate a set of algorithms. Both the SPS and the orchestrated algorithms adhere to a *problem solver interface*. The interface comprises a single function, *solve*, which works iteratively. It may repeatedly request more information about the problem before generating a final result. The function takes the current problem iteration (from $\mathbb{P}_{it}$) and state (from $\mathbb{S}$) to compute the next state:

$$solve: \mathbb{P}_{it} \times \mathbb{S} \rightarrow \mathbb{S} \tag{1}$$

$\mathbb{S}$ contains triplets of the form $(as,req,res) \in AS \times REQ \times RES = \mathbb{S}$, which hold the actual problem solver state (from AS), a request to demand more information (from REQ), and a result for the current problem iteration (from RES). $\mathbb{P}_{it}$ refers to iterated problems:

$$\mathbb{P}_{it} = \mathbb{P} \times \mathbb{H}, \tag{2}$$

comprising the initial problem (from $\mathbb{P}$) and a *request history* $H \in \mathbb{H}$, which is a sequence $H = h_1,\ldots,h_n$ with $h_i \in (REQ,ANS)$, i.e., it is a list of issued requests from REQ and corresponding answers from the set of possible answers, ANS, comprising additional information about the problem (like additional data points of a time series). Note that the different iterations might be required, as in many cases not all information are available from the beginning. Hence, while the final result completely depends on the initial problem and the following answers, the iterations are required to request those answers.

In case of steady state estimation, the initial problem is a time series and the problem solver is a specific estimator. In each iteration, the estimator tries to estimate the steady state statistic. To avoid recalculations, it may store runtime information like the running mean or the variance of the time series in its solver state $as \in AS$. If more data points are required for an estimate, it requests them by setting a boolean request variable to $true$, i.e., $REQ = \{true, false\}$. In that case it also declares the result to be undefined ($\perp$), otherwise it will be the estimated steady state statistic, i.e., $RES = \mathbb{R} \cup \{\perp\}$. As long as the request is $true$, additional data points are generated, e.g., by simulation, and added to the time series. Then, the next iteration of the solution procedure is triggered. This procedure, depicted in Figure S1 of File S1, is executed by an additional management component. It continues until the estimator's request is $false$ and the desired steady state statistic, e.g., the mean, can be estimated.

A more formal description of the problem solver interface is provided in Text S1 of File S1. Many sub-tasks of simulation experiments, e.g., model execution or parameter optimization, are executed in an iterative manner and could thus be controlled

through this interface. The synthetic problem solver (SPS) is a realization of this interface that supports composition. It is formally defined in the next section.

2.2.1 Definition. A synthetic problem solver SPS has the following structure:

$$SPS = \langle A, \mathcal{F}_P, \mathcal{F}_S, \sigma, \kappa \rangle \quad (3)$$

i.e., it is defined by a set of problem solvers (A), sets of problem feature and state feature extractor functions ($\mathcal{F}_P$ and $\mathcal{F}_S$), a selection function σ, and a composition function κ. In the following, we define the different components and their interplay. As with the discussion of existing schemes for algorithm composition (see Section 2.1.1), we base our formal definitions on the notation introduced by Rice [18].

Let A be the set of available problem solvers (algorithms) that implement the described problem solver interface. To realize a decision making procedure on problem solvers, a retrieval of relevant features of those solvers and the problem at hand is required. For example, during a steady state estimation with multiple baseline estimators, problem features might characterize the time series, whereas solver features might represent the results of the estimators when applied to that time series. Therefore, departing from Rice's original definitions, we define two kinds of feature extraction functions. A *problem feature extractor* $F_P : \mathbb{P}_{it} \rightarrow \mathbb{F}_P$ extracts a solver-independent feature $f_p \in \mathbb{F}_P$ from the iterated problem, whereas a *state feature extractor* $F_S : S \rightarrow \mathbb{F}_S$ extracts a solver-*dependent* feature $f_s \in \mathbb{F}_s$ from the state $s \in \mathbb{S}$ of a solver $a \in A$. For instance, in the case of steady state estimation, a problem feature extractor extracts variance and range from a request history that represents the generated time series segments. A state feature extractor could return a boolean that indicates whether an estimator detected the end of the warm-up phase. $\mathcal{F}_P$ denotes the sets of available problem feature extractors, $\mathcal{F}_S$ denotes the set of available state feature extractors.

In some cases, it is not sufficient to just consider the features of the current iteration. For instance, if a problem solver always produced bad results during previous iterations, this could be relevant for decision making. Therefore, we introduce a *feature history* H_F containing all problem and state features that have been previously extracted (note that the feature history is different to the request history of the problem solver interface definition (Equation 2) as it is part of the problem solver state rather than its input). It can be formally described by:

$$H_F = h_1^f, \ldots, h_r^f, \quad (4)$$

where $h_i^f \in \mathbb{F}_P^n \times \mathbb{F}_S^m$, with $n = |\mathcal{F}_P|$ and $m = |\mathcal{F}_S| \cdot |A|$. Hence, the feature history is essentially a list of r tuples, where r is the number of executed iterations and each tuple contains the problem and state features that have been extracted during the corresponding iteration. This allows a decision-making component to consider the entire history of the ongoing problem solving procedure, similar in spirit to the history of the AOTA framework [31].

After the features have been extracted, the decision making process can be executed. Two functions are responsible for this, realizing base-line solver selection and composition. Both are defined upon the set of all possible feature histories, $\mathbb{H}_F$:

1. A *selection function* $\sigma : \mathbb{H}_F \rightarrow 2^A$ decides which (non-empty) set of base-line solvers from A is applied to the current problem iteration.

Table 1. Algorithm 1: Pseudo-code for the solve function of a synthetic problem solver.

1 Given: synthetic problem solver $SPS = \langle A, \mathcal{F}_P, \mathcal{F}_S, \sigma, \kappa \rangle$
2 Input: problem $p_{it}^i \in \mathbb{P}_{it}$, input state $s^i \in \mathbb{S}_{synth}$ (where i is the current iteration)
3 Output: output state $s^{i+1} \in \mathbb{S}_{synth}$
4
5 //extract problem features
6 $E_P \leftarrow \bigcup_{F_P \in \mathcal{F}_P} F_P(p_{it}^i)$
7
8 //select baseline solvers
9 $Sel \leftarrow \sigma(s^i.as.H_f, E_P)$
10
11 //apply selected baseline solvers and retrieve successor states
12 $Suc \leftarrow \{a.solve(p_{it}^i, s^i.as.s_a) \mid a \in Sel\}$
13 $\cup \{s^i.as.s_a \mid a \in A \setminus Sel\}$
14
15 //extract state features from successor states
16 $E_S \leftarrow \bigcup_{F_S \in \mathcal{F}_S, s \in Suc} F_S(s)$
17
18 //append current iteration $(i+1)$ to feature history
19 $H_f' \leftarrow (s^i.as.H_f, (E_P, E_S))$
20
21 //compose next iteration
22 $(res, req) \leftarrow \kappa(H_f')$
23
24 //construct new algorithm state
25 $s^{i+1} \leftarrow ((H_f', Suc), res, req)$

It implements the problem solver interface (see Equation 1).The pseudo-code assumes that problem and state features, i.e., elements from $\mathbb{F}_P$ and $\mathbb{F}_S$, can be collected in sets. The dot-notation, as in $s^i.as.H_f$ (line 9), refers to certain sub-elements of a tuple, in this case the feature history H_f, which is part of the state component as (see Equation 5).
doi:10.1371/journal.pone.0091948.t001

2. A *composition function* $\kappa : \mathbb{H}_F \rightarrow RES \times REQ$ composes the overall results of the selected base-line solvers and generates further requests.

For steady state estimation, a selection function σ could select all estimators that have not yet detected the end of the warm-up phase. Likewise, a composition function κ could request further data points until the majority of estimators has detected an end of the warm-up phase, and then average their estimates to calculate the overall result.

The actual state space (AS) of the synthetic problem solver consists of the feature history and the base-line solver states, so that its overall state $\mathbb{S}_{synth}$ is defined as

$$\mathbb{S}_{synth} = \{(as, res, req) \mid as \in \mathbb{H}_F \times \prod_{a \in A} AS_a, res \in RES, req \in REQ\} \quad (5)$$

Each base-line solver could have a similar state, i.e., synthetic problem solvers can be nested.

2.2.2 Implementation. We can now implement the solve function of the generic interface (see Equation 1) for a synthetic problem solver $SPS = \langle A, \mathcal{F}_P, \mathcal{F}_S, \sigma, \kappa \rangle$. Algorithm 1 in Table 1 shows the pseudo-code. It starts with extracting the set E_P of

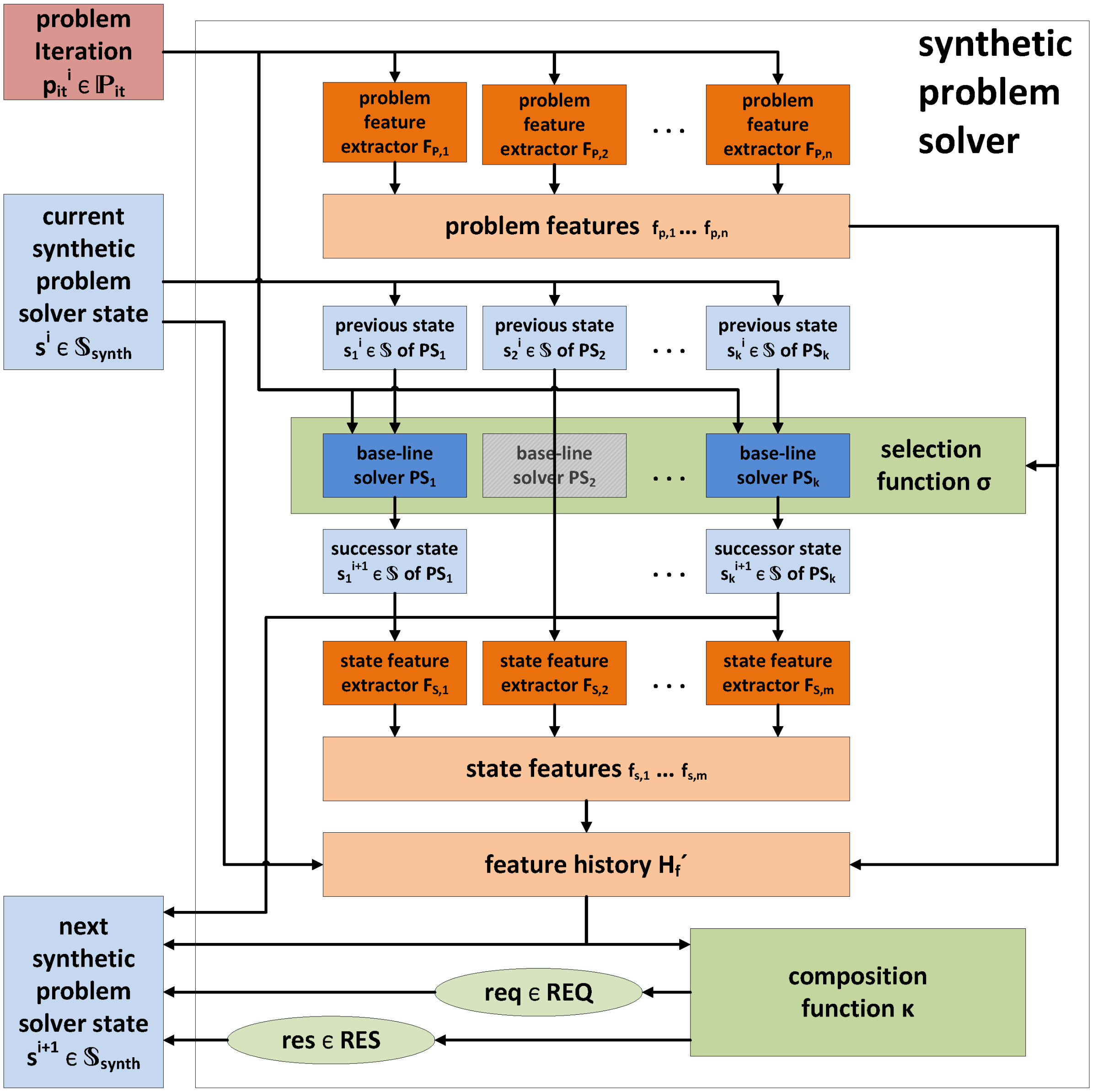

Figure 1. The function solve of the SPS. It receives a problem iteration $p_{it}^{i} \in \mathbb{P}_{it}$ (Equation 2) and the current SPS state $s^{i} \in \mathbb{S}_{synth}$ (Equation 5). At first, the problem feature extractors $F_P \in \mathcal{F}_P$ extract relevant features from p_{it}^{i} (cf. line 6 in Table 1). Then, the selection function σ selects suitable problem solvers for the current problem iteration, p_{it}^{i} (cf. line 9). The selected solvers operate on their previous state $s_1, \ldots, s_k \in \mathbb{S}$ and are applied to p_{it}^{i}, which results in a successor state $s_i' \in \mathbb{S}$. The successor states $s_1' \ldots, s_k' \in \mathbb{S}$ are stored in the SPS successor state s' (l. 25), for the next iteration of solve. Furthermore, they are forwarded to the state feature extractors $F_S \in \mathcal{F}_S$ (l. 16). Note that state feature extractors may only be applicable to certain problem solver states. For instance, some steady state estimator could store a specific test statistic in its state. A corresponding feature extractor would first check compatibility, and then either extract the statistic or return no feature. The features of the current iteration are appended to the feature history H_F (cf. l. 19). The updated feature history H_F' is forwarded to the composition function κ, which creates a result of the current iteration ($res \in RES$), and a request for further information about the problem ($req \in REQ$, cf. l. 22). Finally, result and request are stored in the SPS successor state (cf. l. 25), which is returned and can be used to compute the next iteration of solve.
doi:10.1371/journal.pone.0091948.g001

problem features (see line 6, Algorithm 1 in Table 1). Then, the function σ selects suitable problem solvers, based on the extracted problem features E_P and the feature history H_f (line 9). Afterwards, the solve method of the selected base-line solvers is applied to retrieve their successor states (line 12), while the states of the other base-line solvers remain unchanged (line 13). State features are extracted from the successor states, resulting in the set E_S of extracted state features (line 16). Problem and state features are appended to the feature history H_f, leading to a successor feature history H_f' (line 19) that is used for composing the next result and request of the SPS by applying the composition function κ (line 22). Finally, the successor state s' is created by combining feature history, base-line solver states, result, and request (line 25). Figure 1 shows the data flows during an execution of solve.

2.2.3 An Example SPS for Steady State Estimation. As mentioned before, simulation-based steady state estimation works on time series. In each iteration, an additional part of a time series is created by simulation and investigated by the estimator, i.e.,

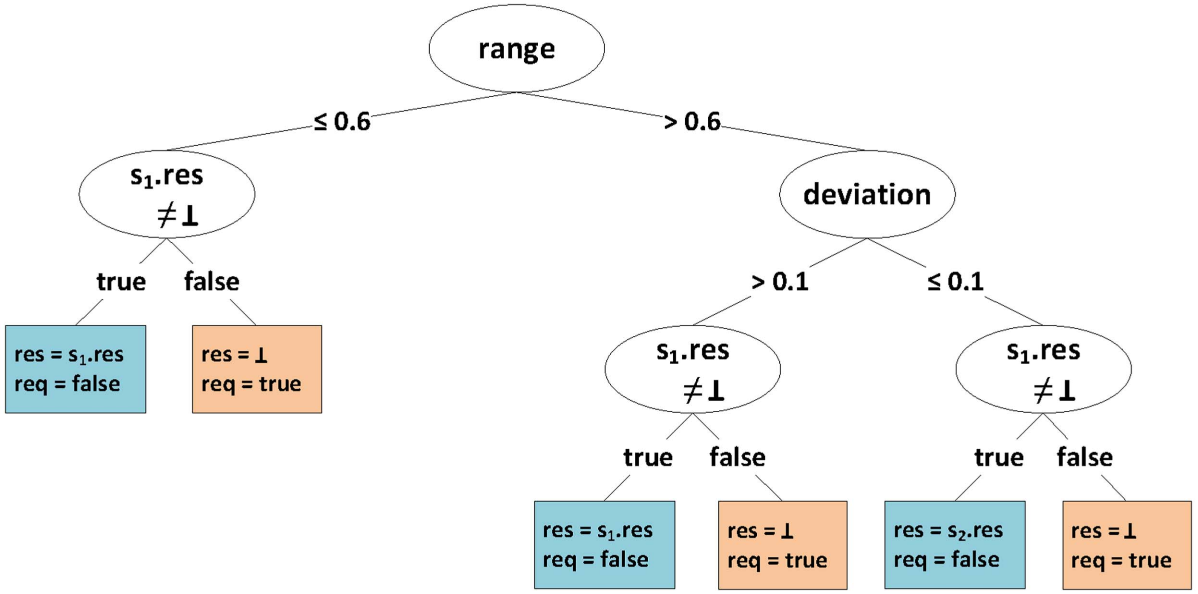

Figure 2. Example decision tree representing the function κ. It is traversed from top to bottom until a leaf is reached, and works on both problem and state features. At first, the (relative) range is considered: if it is below or equal to 0.6, the left sub-tree is selected, otherwise the right one. In the left sub-tree, a decision is made depending on the state feature of estimator a_1, by determining whether it could estimate a steady state mean, stored in its state, i.e., $s_1.res \neq \perp$. In the right sub-tree, the standard deviation is requested. If the deviation falls below 0.1, the state feature of estimator a_1 is considered, otherwise that of estimator a_2 is used. The leaves of the tree denote result and request of the SPS instance. Depending on problem and state features, κ either requests more data points from the simulation or returns the result of the algorithm that is considered more suitable for trajectories with the given features (see line 22 in Table 1).
doi:10.1371/journal.pone.0091948.g002

$\mathbb{P}_{it} = \mathbb{R}^n$. The result is an estimate of some steady state statistic, in our case the steady state mean. Therefore, $RES = \mathbb{R} \cup \{\perp\}$ and $\perp$ again denotes that no steady state mean could be estimated. The request is a boolean value denoting whether additional data are required for estimation, hence $REQ = \{true, false\}$.

Our sample $SPS = \langle A, \mathcal{F}_P, \mathcal{F}_S, \sigma, \kappa \rangle$ uses two base-line problem solvers, i.e., $A = \{a_1, a_2\}$, where a_1 is the MSER steady state estimator [10] and a_2 is Schruben's test steady state estimator [8] (this is a very brief example; we present a more realistic case study in Section 'Experiments: A Synthetic Steady State Estinator'). $\mathcal{F}_{\mathcal{P}}$ contains two problem feature extractors, one for the standard deviation and one for the range, i.e., the distance between minimum and maximum value, of the time series. $\mathcal{F}_S$ contains a state feature extractor that retrieves the estimated steady state mean from the algorithms in A, stored in their states, which may be $\perp$ to denote that none was found yet. The selection function σ is trivial, it always selects both algorithms: $\sigma(H_F) = \{a_1, a_2\}$. The composition function κ is based on a decision tree (e.g., [47]), which is generated from previously collected performance data, where a_1 and a_2 have been applied to representative problems. Figure 2 shows a hypothetical decision tree that could result from such a training process.

2.3 Integration of Synthetic Problem Solvers in James II

2.3.1 Plugin -based Realization. The process of SPS creation, deployment, and usage should be automated as much as possible. Our prototype is based on the open-source modeling and simulation framework James II [15,48] (See http://jamesII.org).

We chose James II as base for the implementation of our concept, as its plugin structure allows a separated and flexible application of algorithms. Furthermore, James II is based on Java, a very popular, platform-independent programming language including a static type-system that is beneficial for the treatment of different algorithm types by maintaining well-defined interfaces. However, our concept can be realized for any simulation system or programming language providing plugins that can be analyzed and applied by reasoning mechanisms.

In James II, algorithms and data structures that are encapsulated in plugins are managed by a central registry. The registry distinguishes plugins based on their *plugin type*, which corresponds to the functionality they offer. Broadly speaking, plugins of a certain plugin type provide alternative implementations of a particular Java interface. James II allows to add new plugins and new plugin types. Both are declared in XML files, loaded during start-up, and can be discovered at runtime, by querying the central registry. This makes it easy for developers to re-use and extend the functionality of the framework.

The prototype of our synthetic problem solver $SPS = \langle A, \mathcal{F}_P, \mathcal{F}_S, \sigma, \kappa \rangle$ (see Equation 3) is based on the following plugins and plugin types:

1. A: a list of problem solver algorithms, i.e., plugins of type *problem solver*. The corresponding Java interface declares the solve function (see Equation 1).
2. $\mathcal{F}_P, \mathcal{F}_S$: lists of problem feature extractors and state feature extractors, i.e., plugins of type *feature extractor*. The distinction between problem and state feature extractors is maintained by defining the type of object to which an extractor can be applied.
3. σ, κ: selection and composition function are implemented by plugins of type *result composer*. The two functions are often related (see Section "Mapping Synthetic Problem Solvers to Existing Composition Schemes"), so we combine them to a single component that fully determines the composition.

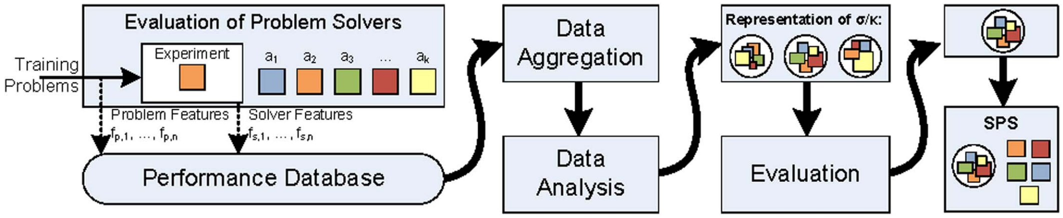

Figure 3. Scheme for the generation. A multi-step procedure allows to automatically generate suitable representations of the σ and κ functions. The procedure involves simulation experiments, data recording, data aggregation, and data analysis. Then, potential representations of σ and κ, e.g., decision trees of various forms, are evaluated against new data, with methods like cross-validation. Finally, the best-performing representations of σ and κ are deployed into an SPS instance.
doi:10.1371/journal.pone.0091948.g003

A, $\mathcal{F}_P$, and $\mathcal{F}_S$ contain predefined plugins, but the composition logic depends on the characteristics of the problems to be solved (see Section 2.1.1). Hence, users should be able to (re-)generate σ and κ automatically. This is described in the following.

2.3.2 Automatic Generation of Selection and Composition Functions. The functions σ and κ of an SPS instance can realize various kinds of composition (see Section "Mapping Synthetic Problem Solvers to Existing Composition Schemes"). They are typically generated from empirical data, by considering the performance of base-line solvers on previously encountered problems. We assume that a set of such *training problems* is available.

Figure 3 shows an overview of the automatic generation process. At first, the base-line problem solvers in A are applied individually to the given training problems. Both problem features and state features are recorded during this evaluation. State features can also include performance measurements, such as memory consumption or execution time. The features are extracted automatically and written to a dedicated performance database [49], which is integrated into the experimentation layer of James II [16]. This makes it easy to conduct large-scale performance evaluations with various experiment designs, so that even large sets of training problems can be analyzed conveniently.

Often, an additional data aggregation step is required. For example, generating a κ function for an algorithm ensemble requires to aggregate the data tuples of all individual problem solvers (see Section 2.4.3). Aggregation may also be necessary if either algorithm performances or training problems are stochastic.

Given the aggregated data, we can now generate different representations of the σ and κ functions. This 'Data Analysis' step (see Figure 3) is handled by plugins. For example, one plugin could implement a machine learning algorithm to create decision trees like the one shown in Figure 2. Several machine learning algorithms are already available as plugins, e.g., implementations from the WEKA toolkit [50].

Then, the representations of σ and κ are evaluated on previously unseen problems. This step ensures, for example, that a learning method is indeed suitable to the domain at hand, e.g., by estimating its prediction error on previously unseen problems. Like the data analysis step, this procedure is fully customizable via plugins. In a final step, the best-performing representations of σ and κ are incorporated in a new SPS instance. We describe its deployment to James II in the next section.

2.3.3 Deployment to the James II Registry. The result of the process depicted in Figure 3 is an instance of the synthetic problem solver, which needs to be deployed as a new plugin to the James II registry. However, the way in which James II plugins are created and loaded is rather static. Typically, plugins are declared in XML files that contain relevant Java class names and some pre-defined meta-data, e.g., regarding parameters [15]. An SPS instance, however, is not defined by a class, but instead by specific *instances* of its base-line problem solvers (A), its feature extractors ($\mathcal{F}_P$ and $\mathcal{F}_S$), and its selection and composition functions (σ and κ). Therefore, we extend the registry to support a more flexible definition of plugins.

SPS instances are wrapped in so-called *synthetic plugins*, which are handled by an additional management component. Synthetic plugins are stored to a local file, which is read during start-up. While they are kept in a dedicated data structure, i.e., separated from the other James II plugins, queries to discover a plugin of a certain type now also consider all synthetic plugins of that type. This process is transparent to application code, i.e., an SPS instance can be applied throughout James II without code changes.

After an SPS has been trained and deployed (typically, by an expert for the problem domain or the algorithms at hand), users do not have to care about selecting the best suited algorithm for a problem, or how to apply trained compositions. Instead, they simply use the deployed synthetic plugin, which returns an SPS instance comprising the composition. Synthetic plugin and SPS instance are thus used similarly to the standard plugins and algorithms offered by James II.

Synthetic plugins may provide additional meta-data that is important for selecting between them. In case of steady state estimation, this could include the statistical properties of the time series used for training.

2.4 Mapping Synthetic Problem Solvers to Existing Composition Schemes

We now elaborate how the SPS can be configured to realize the composition approaches discussed in Section 2.1.1. The simplicity of these mappings makes us confident that this approach could be useful for many other problems encountered in computational systems biology.

2.4.1 Algorithm Selection Problem. The first element of an $SPS = \langle A, \mathcal{F}_P, \mathcal{F}_S, \sigma, \kappa \rangle$, A, corresponds to the set of algorithms $\mathbb{A}$ in the algorithm selection problem (see Section 2.1.1). However, each algorithm in A has to comply with the problem solver interface (see Equation 1). The problem feature extractors in $\mathcal{F}_P$ are analogous to feature extractors in the ASP, but $\mathcal{F}_P$ would also include a problem feature extractor that returns user criteria. The set of state feature extractors, $\mathcal{F}_S$, contains a single extractor that retrieves the overall results from the state of the selected base-line problem solver. As only one base-line problem solver shall be selected and used, the selection function σ picks a single element from A and thus realizes a selection mapping for the ASP. Typically, σ is generated by analyzing algorithm performance on training problems. This analysis relies, at least implicitly, on some measure of performance, which is explicitly considered by the ASP

but has no direct counterpart in the SPS. The composition function κ returns the results extracted from the state of the selected base-line problem solver.

As a sample setup, consider a set A of stochastic simulation algorithms (SSAs) for chemical reaction networks [51]. SSA performance strongly depends on the given model, so that automatically selecting a suitable algorithm can considerably improve execution time [12]. The problem space $\mathbb{P}_{it}$ contains all chemical reaction networks that can be simulated with SSAs. Problem feature extractors in $\mathcal{F}_P$ may retrieve the number of distinct species and reactions from a model, or a measure of its stiffness. An additional user criterion could, for example, account for approximative SSA variants and specify an acceptable trade-off between execution speed and accuracy. The state feature extractor in $\mathcal{F}_S$ would retrieve the generated simulation trajectory from an SSA state, and the selection function σ could be generated via supervised learning on previously recorded execution times. A similar approach was pursued in [52] for spatial SSAs. The composition function κ would return the generated simulation trajectory to the user.

2.4.2 Algorithm Portfolios. If an $SPS = \langle A, \mathcal{F}_P, \mathcal{F}_S, \sigma, \kappa \rangle$ realizes an algorithm portfolio, the portfolio elements correspond to the algorithm set A. Problem features are typically neglected for portfolio construction, so $\mathcal{F}_P$ would be empty. $\mathcal{F}_S$ would contain at least two state feature extractors: one to retrieve the current solution from a base-line problem solver state, and one to retrieve its requests. The selection function σ would select all portfolio members for execution, whereas the composition function κ would aggregate the base-line problem solver's results and requests based on their predefined portfolio weights.

Algorithm portfolios can be applied, for example, to solve hard optimization problems [24] in systems biology [53]. In case of simulation-based optimization, each base-line problem solver could be an optimization approach that requests new points in a model's parameter space to be evaluated. The κ function could aggregate all requests based on portfolio weights, e.g., to let more promising optimization algorithms evaluate more data points. Dynamic portfolios, where algorithm weights are adapted during iterations, can be mapped to an online adaptation scheme (see Section 2.4.4).

2.4.3 Algorithm Ensembles. The realization of ensembles by the SPS is quite similar to algorithm portfolios. The only difference is that the composition function κ now realizes an ensemble function that takes into account all base-line problem solver results. This may also require that the original problem $p \in \mathbb{P}$ is stored as a problem feature, so that it can be accessed by κ.

Our SPS setup described in Section 3.1 is a concrete example of such an approach. Machine learning approaches can be improved in similar ways [27,29], and can be applied to various problems in systems biology (e.g., see [54]).

2.4.4 Online Adaptation. All of the above schemes can be adapted to work in an online fashion, leading to dynamic algorithm selection, dynamic algorithm portfolios, and dynamic algorithm ensembles. In general, adaptive behavior in the SPS can be realized by defining problem and state feature extractors that extract metrics regarding the solution progress. Such data is then stored to the feature history H_f, so that the σ and κ functions can access them. Data to be accessed by the solvers itself can be added to the answer-request history by the composition function κ, so that it becomes part of the next problem iteration $p \in \mathbb{P}_{it}$.

For example, an adaptation scheme like AOTA (see Section 2.1.1) can be realized this way. AOTA's history corresponds to the feature history $\mathbb{H}_F$. The composition function κ could conduct solver-specific performance predictions and could then store the results. During the next iteration, the base-line problem solvers can access those results, now part of the iterated problem $p \in \mathbb{P}_{it}$, and use computing resources accordingly.

Approaches that adaptively reconfigure a simulator at runtime (e.g., [33,42]) can be mapped to this scheme as well. The selection function σ would realize the online learning algorithm and select the most suitable simulator for the next part of the simulation task, while the composition function κ would simply store the last model state (extracted from the selected base-line problem solver) to the answer-request history, so that it can be accessed by the next base-line problem solver.

Experiments: A Synthetic Steady State Estimator

To illuminate the effectiveness of the developed SPS infrastructure for supporting simulation studies in systems biology, we apply it to steady state estimation [55, p. 96], i.e., we aim at estimating the mean of a time series after its warm-up phase (also called initial transient or initialization bias) is finished. This application area is of particular interest for a composition of base-line problem solvers, as Assmussen et. al. [57] proved that no universal solution exists for finding the end of the warm-up phase, i.e., no solution *always* yields correct results (independently of specific trajectory features). Hoad et. al. [56] affirmed this finding for practical applications, which conforms to our experience when conducting experiments with different steady state estimators (Section Text S2 of File S1). Hence, even an expert for steady state estimation cannot easily predict which steady state estimator performs best on a given problem. The expert can, however, provide the required information for generating an SPS instance that performs well on the given problem's features, and can be easily applied by a non-expert user.

We evaluate our approach by composing ten steady state estimators to an SPS instance and then testing it with time series generated by several biochemical models. Performance measures focus on accuracy and robustness of the estimators. Time efficiency is not considered to be an issue, as the bottleneck in simulation experiments usually resides in the model execution that generates the time series and not in their analysis. However, as the generation of time series is costly, we also investigate the data efficiency of the tested estimators.

3.1 Experiment Setup

We describe the elements of the $SPS = \langle A, \mathcal{F}_P, \mathcal{F}_S, \sigma, \kappa \rangle$ that have been realized for creating a synthetic steady state estimator.

3.1.1 Base-line Problem Solvers (A). We implemented the following steady state estimators as base-line problem solvers:

1. MSER: identifies the warm-up phase's end by deleting initial observations to the point that provides the minimal MSER statistic [10].
2. Euclidean Distance: divides a time series into vectors and normalizes them. If all vectors of a sequence are close enough to the unit vector, the end of the warm-up phase has been detected [11].
3. Goodness of Fit: divides a time series into batches, counts the amount of values below and above the mean for each batch, and performs a Chi-Square test on the resulting histograms to identify the warm-up phase [58].
4. Balancing Mean: counts the amount of values above and below the mean of a time series. If the difference between both counts is below a given threshold, the end of the warm-up phase has been detected [6].

5. Running Mean: assumes the end of the warm-up phase as soon as the change in the running mean falls below a given threshold [58].
6. Batch Mean: divides a time series into batches and the batches into two groups. Assumes the end of the warm-up phase as soon as the distributions of the variances for the two groups are close enough [9].
7. Crossing Mean: counts the crosses of a time series and its running mean. As soon as a given amount of crosses occurred, the end of the warm-up phase is assumed [7].
8. Stop Crossing Mean: counts the crosses of time series and running mean. As soon as no crosses happened for a given length, the end of the warm-up phase is assumed.
9. Schruben's Test: estimates the stationarity of a time series to identify its warm-up phase [8].
10. Moving Windows: moves a window through a time series and updates the mean according to the values inside the window. If the standard deviation falls below a given threshold, the warm-up phase has been detected [59].

Methods 1–3 have been proposed by [57] for automated experimentation, as they do not require a careful configuration. The others have been proposed during the last four decades and are still in use, which illustrates the difficulty of selecting a suitable steady state estimator for a given problem. We realized all methods as plugins of James II, and use them with their default parameters. The result of each problem solver is the estimated steady state mean, or $\perp$ if no steady state mean could be estimated. Hence, $RES = \mathbb{R} \cup \{\perp\}$. The request is a boolean value denoting whether additional data points are required for estimation, hence $REQ = \{true, false\}$.

3.1.2 Problem Feature Extractors ($\mathcal{F}_P$). The feature extractors shall characterize two key aspects of steady state mean estimation on a time series : the amount of noise and the shape of the initialization bias. We implemented the following time series feature extractors in James II:

1. Count of values above sample mean, to measure the amount of values increasing the positive bias.
2. Count of values below sample mean, to measure the amount of values increasing the negative bias.
3. Count of values below sample mean subtracted from those above sample mean, to measure whether positive or negative values dominate the time series.
4. Maximum positive distance to sample mean, to measure the impact of values increasing the positive bias.
5. Minimum negative distance to sample mean, to measure the impact of values increasing the negative bias.
6. Maximum absolute distance to sample mean, to measure the overall bias.
7. Portmanteau Test [60], to get a measure for the autocorrelation, and therefore an indication whether patterns in the curve are repeated.

Features 1–6 characterize the type of bias (mainly positive or negative) in the time series, feature 7 gives an idea about the noise. We normalized the features in order to scale with different time series sizes. Features 1–3 are divided by the size of the time series and features 4–7 by its value range.

For a better characterization of the initialization bias in the presence of noise, we implemented additional extractors that calculate the above features from a smoothed trend curve of the time series. We used an exponential smoothing algorithm [61] for creating the trend curve.

Previous experiments showed that feature 6 extracted from the time series (not on the trend curve) biased the learning process of the composition function (see Section 3.1.5), leading to overfitted results. Hence, we leave this particular feature extractor out in our experiments, so that we extract $6+7=13$ problem features overall.

3.1.3 State Feature Extractors ($\mathcal{F}_S$). Regarding state features, we are particularly interested in the *detection result* of a steady state estimator, i.e., whether it found the end of the warm-up phase in a time series or not. This detection result is retrieved by a state feature extractor f_d.

The extractor f_d returns 1 if the request element of the given state equals false, i.e., the end of the warm-up phase has been detected, so that a steady state can now be estimated and no further data is required. Otherwise, f_d returns 0 if more data is requested, i.e., the end of the warm-up phase has not been detected yet:

$$f_d(s) = \begin{cases} 1, & \textit{if } s.req = \textit{false} \\ 0, & \textit{otherwise} \end{cases} \tag{6}$$

3.1.4 Selection Function (σ). As no selection of base-line solvers is considered, the selection function returns all given base-line solvers:

$$\sigma(H_F, A) = A \tag{7}$$

3.1.5 Composition Function (κ). We generate a composition function with a wrapper for WEKA's J48, which is an implementation of the C4.5 decision tree algorithm [62]. The basic idea behind decision trees is to identify those attributes (i.e., features) of training set instances that contain most information regarding the class attribute (i.e., end of warm-up phase detected or not). This attribute selection is done recursively, to construct a tree of nodes representing decisions on attributes. However, the used tree differs from the example tree of Figure 2, as it decides on the end of the problem time series ' warm-up phase, instead of deciding on the steady state estimator that shall be used for creating estimation results. This approach offers further opportunities for composition, as the results of different steady state estimators can be combined after the end of the warm-up phase has been found. In the present example, they have been combined by using an equally weighted portfolio of the base-line problem solvers.

To generate training data for the J48, we use a problem generator that creates sample problems. It follows the evaluation approaches of [9], [63], [64], and [57] that generate time series over several successive steps, instead of using an M/M/1 queue as, e.g., [65], [66], and [67] do. The reason for this decision is its straightforward parametrization, which allows for a direct control of several key features (see below). The problem generator has the following structure:

$$G = \langle t, s, l_b, l_o, n, c \rangle \tag{8}$$

It generates time series with bias of length l_b, trend t, and shape s. The noise of the time series is induced by a random number generator and varies between $-n$ and $+n$. The series is of size l_o and the numbers are cross correlated with factor c.

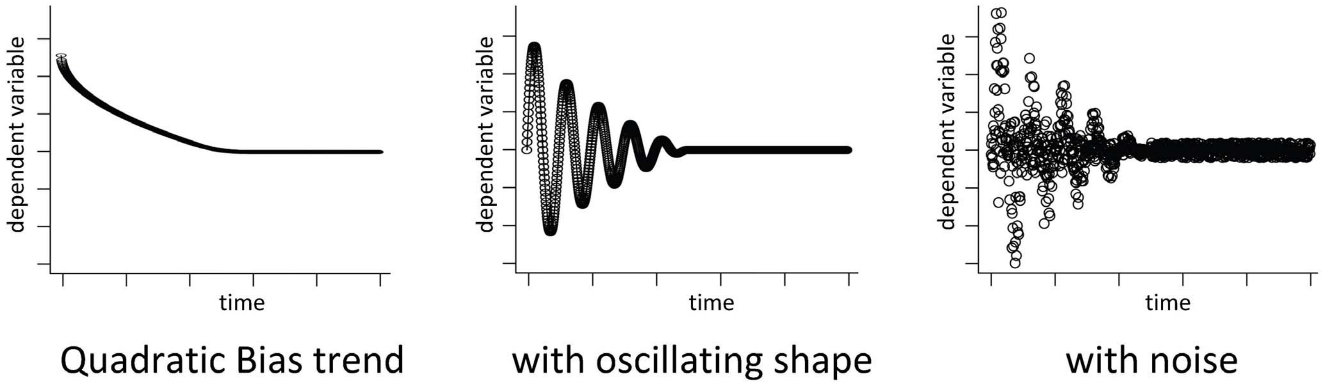

Figure 4. Successive generation of problem time series.
doi:10.1371/journal.pone.0091948.g004

We selected trend and shape to cover the most relevant types of time series, orienting our selection towards previous studies [9,57,63,64]. We pursued a more fine-grained investigation of bias during the warm-up phase, by distinguishing between two dimensions. The first dimension is bias trend t which includes two variants: constant and quadratic. The second dimension is bias shape s, where three variants are considered: a straight line shape, an oscillating shape, and a random shape. By combining the shapes and trends, six types of bias can be created. For instance, a quadratic trend with an oscillating shape means that during the warm-up phase, the time series initial bias oscillates with a quadratically decreasing amplitude, as shown in Figure 4.

In addition to bias, three other factors have been varied in our experiments: noise, cross-correlation, and bias length. We tested bias lengths from 0 to 150 percent of the time series length. Bias length of more than 100 percent allows to investigate the ability of an estimator to handle heavily biased time series. Furthermore, as stochasticity plays a role in steady state estimation, we tested different noise levels to investigate the robustness of the estimators against them. The noise levels range from 0 to 10 percent, where, e.g., 10 percent noise means that a random value between -10 and 10 percent is added to each value of the time series. The noise has been generated with an auto-correlation factor of 0.5 to get more realistic random numbers that are not independent and identically distributed. Finally the length of the time series has been varied between 1,000 and 10,000 time points.

We generated 8,640 problem definitions, each being used to randomly generate 10 time series to tackle stochastic bias. Hence, each of the used steady state estimators has been applied to 86,400 time series.

3.1.6 Problem Solver Evaluation. For simplicity, the steady state estimators used in this study are considered as monolithic entities, i.e., each tested steady state estimator is given with a fixed (default) parametrization. Hence, a distinct consideration of the influence of different parameter settings is not done, but should be the subject of follow-up studies.

We compare the performance of the created SPS instance to those of the base-line problem solvers, by applying them to real simulation data. To do so, we execute five π-Calculus models [68] that produce time series as input for the steady state estimators.

The models are examples models for the *Stochastic Pi Machine (SPiM)* [17]:

- MgCl2: representing the behavior of a $MgCl_2$ solution. The time series contains the counts of the Mg particles over time.
- NaCl: representing the behavior of a $NaCl$ solution. The time series contains the counts of the Na^+ particles over time.
- KNa2Cl: representing the behavior of a KNa_2Cl solution. The time series contains the counts of the Na^+ particles over time.
- HCl: representing the behavior of a HCl solution. The time series contains the counts of bound H particles over time.
- rNHCOR: representing the synthesis reaction of an rNHCOR amide. The time series contains the counts of the $rNHCOR$ particles over time.
- MAPK: representing a simplified MAPK cascade. The time series contains the counts of the KBP cells over time.

In addition to these 'toy' models we test the steady state estimators with output generated by an ML-rules implementation [69] of the T cell receptor (TCR) signaling model by Lipniacki et. al. [3]. The model has been designed to investigate the stochastic influences and bistability in TCR dynamics. It is focused on the MAP kinase ERK, the amounts of which are converted into a time series and used as input for the steady state estimators.

For assessing the steady state estimators, we focus on accuracy, robustness, and data efficiency. Efficiency with respect to required computational resources (computation time, memory, etc.) is of less importance compared to the time effort that is required to generate time series by simulation (each simulation run took at least five minutes, whereas any estimator application took less than a second, in our example experiment). We investigate two performance measures that are of practical relevance for applying steady state estimators, i.e., the ability to find the end of the warm-up phase and the deviation between estimated and real steady state mean.

The investigation of additional measures could be of interest for certain applications, e.g., the ability to cope with different noise levels in the time series. A comparative analysis of additional performance aspects, e.g., via principal component analysis [70], is subject to future work.

Each time series of the test models is processed by the problem solvers in maximal 15 iterations, i.e., the time series are divided into 10 chunks. The first 5 chunks comprise the first time points of the time series, until the *ideal truncation point* (The ideal truncation point is calculated according to the *mean squared error (MSE)* based algorithm proposed by Wilson and Pritsker [67]) is reached, i.e., the warm-up phase is equally divided into the first 5 chunks. The following 10 chunks comprise the next 10 segments of the time series (after the truncation point).

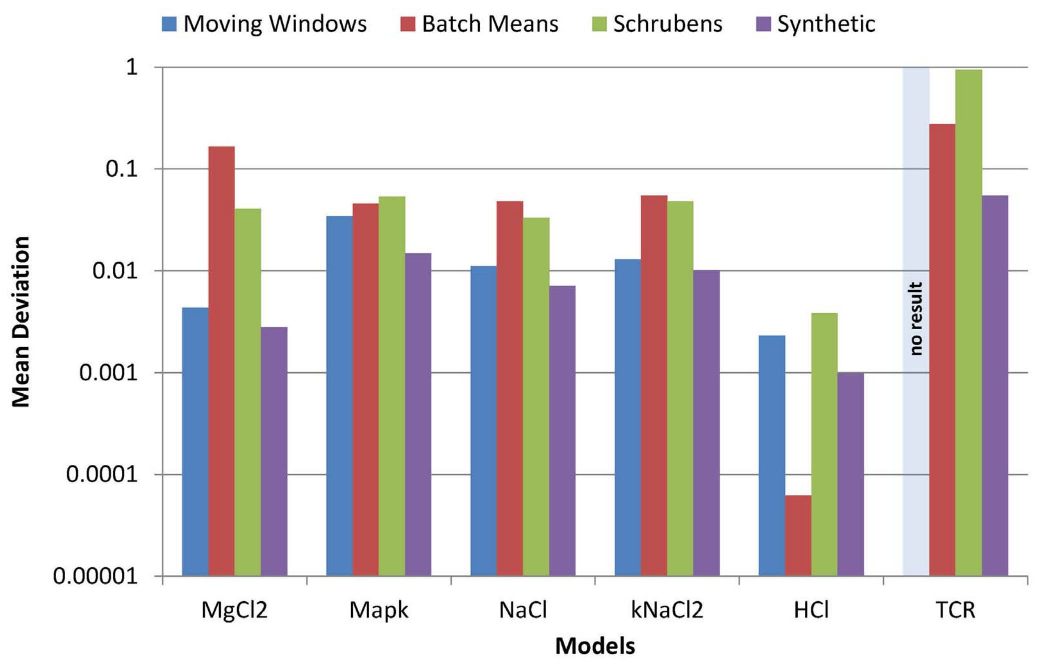

Figure 5. The accuracy of the steady state estimators. It is measured as relative distance between real and estimated steady state on the time series produced by the evaluation models. Note that the deviation axis is scaled logarithmically.
doi:10.1371/journal.pone.0091948.g005

This kind of input data allows us to investigate the accuracy, robustness, and data efficiency of steady state estimators. We measure accuracy by calculating the distance between the real steady state mean and the mean estimated by the steady state estimator. For measuring the amount of required data, we count the amount of chunks until the estimator produces a result, where estimators are considered robust if they detect the end of the warm-up phase after the first 5 chunks (where the warm-up phase actually has ended), and data efficient if they detect it soon after the 5th chunk.

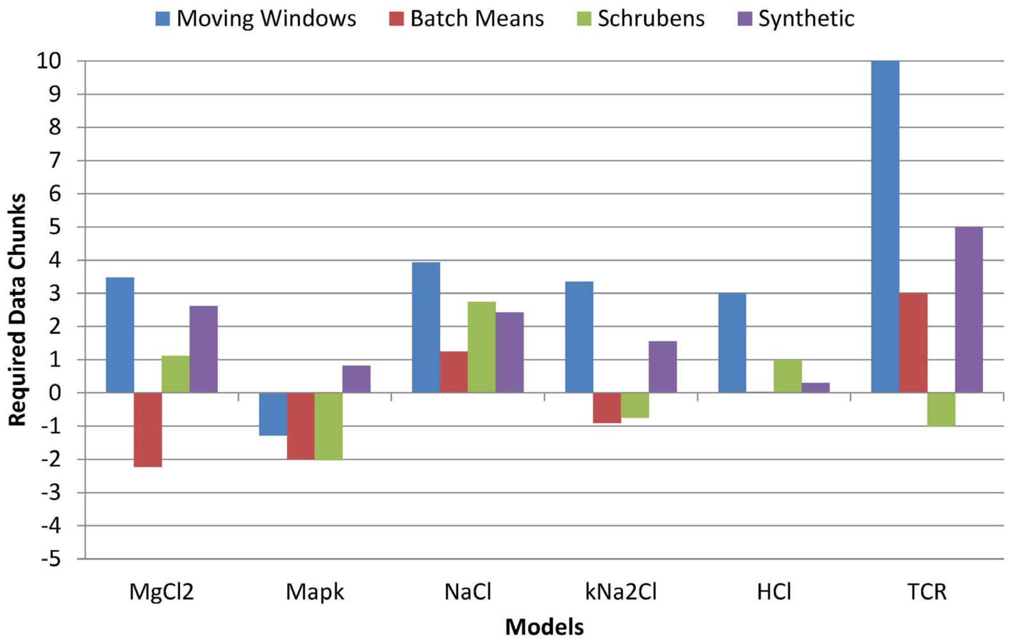

Figure 6. The number of required iterations the steady state estimators needed. for producing an estimate on the time series produced by the evaluation models. Negative values correspond to chunks of data points before the ideal truncation point. Positive values correspond to chunks of data points after the ideal truncation point.
doi:10.1371/journal.pone.0091948.g006

Each evaluation run (i.e., application of a problem solver onto a set of time series chunks) has been repeated 100 times to gain reliable results.

3.2 Evaluation Results

The accuracy of problem solvers is depicted in Figure 5. From the base-line problem solvers, we only present the results of the Moving Window, Batch Means, and Schruben's Test steady state estimators, as they performed best among the 10 tested base-line steady state estimators during the evaluation experiment. The results of the remaining estimators is depicted in Table S1 of File S1. The results of the rNHCOR model are not discussed, as none of the executed estimators was able to estimate a steady state on 15 time series chunks generated by this model.

The SPS instance is the most accurate estimator on all time series but those generated by the HCl model (where it is the second best). On time series generated by this model, all estimators produce very accurate results with less than 1 percent deviation from the real steady state mean. Hence, the worse performance of the SPS instance could be explained by statistical fluctuations resulting from the closeness of the different estimates.

Figure 6 shows the required chunks of data points until a steady state could be estimated. Negative values correspond to the first 5 chunks of the time series (-5 being the first chunk, -4 the second etc.), i.e., the corresponding problem solver gave a steady state estimate on time series data before the warm-up phase was finished. Hence, negative values indicate that the problem solver is less robust, as it returns biased results. On the other hand, positive values correspond to the next 10 chunks of the time series (1 being the first chunk after the truncation point, 2 the second etc.), i.e., high positive values indicate that the estimator requires many data points after the ideal truncation points to give an estimate, making it less efficient.

The SPS instance is the only estimator where the number of required chunks is higher than 0 for all models, which shows its robustness. While estimators exist that require less chunks after the ideal truncation point, the SPS is at least the second best estimator in this regard, and hence moderately efficient.

Altogether, the generated SPS instance is the most accurate steady state estimator in our evaluation. It outperforms the other estimators when applied to input data created by all but one model. In addition, it is the most reliable estimator and its efficiency (i.e., required data chunks) is acceptable. While the run time of the SPS instance might be higher than that of a base-line steady state estimator, the impact on overall experiment duration is usually negligible. This is because the generation of time series by simulation typically requires much more computing time than their analysis.

Conclusion

We presented a generic scheme to compose simulation software components for improved performance, and exemplified our approach by composing a steady state estimator that is tuned to the simulation of biochemical models. Our composition approach is based on the notion of a synthetic problem solver, which iteratively applies sub-algorithms to a problem and aggregates their results. SPS instances can be trained on representative sample problems from a given problem domain. With the SPS, users are released from a manual selection of the best suited algorithm for their problems.

The aggregation logic is highly customizable and can be generated automatically, e.g., via machine learning. We implemented an SPS prototype for the simulation framework James II and automated its configuration and deployment, and showed how other composition schemes can be realized as synthetic problem solvers. In our case study, we generated and evaluated an SPS instance for the steady state estimation of biological models.

While we developed all necessary tools for the automatic creation of an SPS, a more intuitive and user-friendly workflow is still work in progress. For this, we are going to rely on the workflow management system WorMS [71]. We also plan to apply our approach to other problem domains, such as cycle detection in time series or simulation-based optimization.

Supporting Information

File S1 Supporting figures and tables. **Text S1**. A Problem Solver Interface. **Figure S1**. Sequence diagram depicting the iterated communication between answer function and a problem solver. **Figure S2**. Example scheme for managing the communication between a simulator producing time series for a steady state estimator. **Text S2**. Base-Line Steady State Estimator Results on the Training Data. **Figure S3**. Mean deviation and success rate of the tested steady state estimators applied on problem data. **Figure S4**. Mean deviation and success rates of the tested steady state estimators applied on time series with different amounts of noise. **Table S1**. Steady State Estimator Evaluation Results on Simulation Data.
(PDF)

Acknowledgments

We thank James Faeder, who suggested the TCR model for evaluating steady state estimators, and Tobias Helms, who implemented the ML-Rules version of this model.

Author Contributions

Conceived and designed the experiments: SL RE. Performed the experiments: SL RE. Analyzed the data: SL RE. Contributed reagents/materials/analysis tools: SL RE. Wrote the paper: SL RE AMU. Software Implementation: SL RE.

References

1. Woods YL, Lane D (2003) Exploiting the p53 pathway for cancer diagnosis and therapy. The Hematology Journal 4: 233–247.
2. Garg A, Xenarios I, Mendoza L, Micheli GD (2007) An efficient method for dynamic analysis of gene regulatory networks and in silico gene perturbation experiments. In: Proceedings of the International Conference on Research in Computational Molecular Biology (RECOMB).
3. Lipniacki T, Hat B, Faeder JR, Hlavacek WS (2008) Stochastic effects and bistability in T cell receptor signaling. Journal of theoretical Biology 254: 110–122.
4. Davidich MI, Bornholdt S (2008) Boolean network model predicts cell cycle sequence of fission yeast. PLoS ONE 3: e1672.
5. Ay F, Xu F, Kahveci T (2009) Scalable steady state analysis of boolean biological regulatory networks. PLoS ONE 4: e7992.
6. Emshoff JR, Sisson RL (1970) Design and use of computer simulation models. The MacMillan Company.
7. Wilson JR, Pritsker AAB (1978) A survey of research on the simulation startup problem. Simula-tion 31: 55–58.
8. Schruben LW (1982) Detecting initialization bias in simulation output. Operations Research 30: 151–153.
9. Cash CR, Dippold DG, Long JM, Nelson BL (1992) Evaluation of tests for initial-condition bias. In: Proceddings of the Winter Simulation Conference. pp. 577–585.
10. White Jr KP (1997) An effective truncation heuristic for bias reduction in simulation output. Simulation 69: 323–334.
11. Lee YH, Kyung KH, Jung CS (1997) On-line determination of steady state in simulation outputs. Computers Industrial Engineering 33: 805–808.

12. Ewald R (2011) Automatic algorithm selection for complex simulation problems. Vieweg+Teub-ner. URL http://dx.doi.org/10.1007/978-3-8348-8151-9. doi:10.1007/978-3-8348-8151-9
13. Leye S, Uhrmacher AM (2010) A exible and extensible architecture for experimental model val-idation. In: Proceddings of the International Conference on Simulation Tools and Techniques (SIMUTools).
14. Ghosh S, Matsuoka Y, Asai Y, Hsin KY, Kitano H (2011) Software for systems biology: from tools to integrated platforms. Nature Reviews Genetics 12: 821–832.
15. Himmelspach J, Uhrmacher AM (2007) Plug'n simulate. In: Proceedings of the Annual Simulation Symposium. pp. 137–143.
16. Ewald R, Himmelspach J, Jeschke M, Leye S, Uhrmacher AM (2010) Flexible experimentation in the modeling and simulation framework JAMES II – implications for computational systems biology. Briefings in Bioinformatics 11: 290–300.
17. Phillips A. Examples in SPiM. Http://research.microsoft.com/en-us/projects/spim/examples.pdf. Accessed 2013 July.
18. Rice JR (1976) The algorithm selection problem. Advances in Computers 15: 65–118.
19. Kaddi C, Quo CF, Wang MD (2008) Quantitative metrics for bio-modeling algorithm selection. In: International Conference on Engineering in Medicine and Biology Society. pp. 4613–4616.
20. Houstis EN, Catlin A, Rice J, Verykios V, Ramakrishnan N, et al. (2000) PYTHIA II: a knowl-edge/database system for managing performance data and recommending scientific software. ACM Transactions on Mathematical Software 26: 227–253.
21. Markowitz H (1952) Portfolio selection. The Journal of Finance 7: 77–91.
22. Xu L, Hutter F, Hoos HH, Leyton-Brown K (2008) SATzilla: Portfolio-based algorithm selection for SAT. Journal of Artificial Intelligence Research 32: 565–606.
23. Huberman BA, Lukose RM, Hogg T (1997) An economics approach to hard computational prob-lems. Science 275: 51–54.
24. Gomes CP, Selman B (2001) Algorithm portfolios. Artificial Intelligence 126: 43–62.
25. Figge F (2004) Bio-folio: applying portfolio theory to biodiversity. Biodiversity & Conservation 13: 827–849.
26. Kitano H (2010) Violations of robustness trade-offs. Molecular Systems Biology 6.
27. Rokach L (2010) Ensemble-based classifiers. Artificial Intelligence Review 33 (1–2): 1–39.
28. Yang P, Yang YH, Zhou BB, Zomaya AY (2010) A review of ensemble methods in bioinformatics. Current Bioinformatics 5.
29. Smith-Miles KA (2008) Cross-disciplinary perspectives on meta-learning for algorithm selection. ACM Computing Surveys 41: 1–25.
30. Gagliolo M, Zhumatiy V, Schmidhuber J (2004) Adaptive online time allocation to search algo-rithms. Technical report, Istituto Dalle Molle di studi sull'intelligenza artificiale.
31. Gagliolo M, Schmidhuber J (2006) Dynamic algorithm portfolios. In: Proceedings of the Interna-tional Symposium on Artificial Intelligence and Mathematics.
32. Sutton RS, Barto AG (1998) Reinforcement learning: an introduction. MIT Press.
33. Helms T, Ewald R, Rybacki S, Uhrmacher AM (2013) A generic adaptive simulation algorithm for component-based simulation systems. In: Proceedings of the Conference on Principles of Advanced Discrete Simulation (PADS). pp. 11–22. URL http://dx.doi.org/10.1145/2486092.2486095. doi:10.1145/2486092.2486095
34. Norvig P, Cohn D (1997) Adaptive software. PC AI 11: 27–30.
35. Mckinley PK, Sadjadi SM, Kasten EP, Cheng BHC (2004) Composing adaptive software. Computer 37: 56–64.
36. IBM (2001). Autonomic computing: IBM's perspective on the state of information technology. URL http://www.research.ibm.com/autonomic/manifesto/. www.research.ibm.com/autonomic/manifesto/autonomic_computing.pdf, Accessed 2013 July.
37. Beckman B (2012) Why LINQ matters: cloud composability guaranteed. Communications of the ACM 55: 38–44.
38. Seacord RC, Mundie D, Boonsiri S (2001) K-BACEE: knowledge-based automated component ensemble evaluation. In: Euromicro Conference. pp. 56–62.
39. Ostertag E, Hendler J, Díaz RP, Braun C (1992) Computing similarity in a reuse library system: an ai-based approach. ACM Transactions on Software Engineering Methodology 1: 205–228.
40. Ferscha A (1995) Probabilistic adaptive direct optimism control in time warp. In: Proceedings of the Workshop on Parallel and Distributed Simulation (PADS). Washington, DC, USA: IEEE Computer Society, pp. 120–129. URL http://dx.doi.org/10.1145/214282.214320. doi:10.1145/214282.214320
41. Das SR (1996) Adaptive protocols for parallel discrete event simulation. Proceddings of the Winter Simulation Conference : 186–193.
42. Meraji S, Tropper C, Zang W (2010) A multi-state q-learning approach for the dynamic load balancing of time warp. Proceedings of the International Workshop on Principles of Advanced and Distributes Simulation (PADS) : 1–8.
43. Boukerche A (2001) An adaptive partitioning algorithm for conservative parallel simulation. In: IPDPS.
44. Minson R, Theodoropoulos G (2007) Adaptive support of range queries via push-pull algorithms. In: Proceedings of the International Workshop on Principles of Advanced and Distributed Simu-lation (PADS). pp. 53–60.
45. Vitali R, Pellegrini A, Quaglia F (2010) Autonomic Log/Restore for advanced optimistic simulation systems. In: Proceedings of the International Symposium on Modeling, Analysis and Simulation of Computer and Telecommunication Systems. MASCOTS '10, pp. 319–327.
46. Higman DJ (1993) Error control for initial value problems with discontinuities and delays. Applied Numerical Mathematics 12: 315–330.
47. Quinlan JR (1986) Induction of decision trees. Machine learning 1: 81–106.
48. Himmelspach J, Ewald R, Uhrmacher AM (2008) A exible and scalable experimentation layer. In: Proceedings of the Winter Simulation Conference (WSC). pp. 827–835.
49. Ewald R, Uhrmacher AM, Saha K (2009) Data mining for simulation algorithm selection. In: Proceedings of the International Conference on Simulation Tools and Techniques. ICST.
50. Machine Learning Group at the University of Waikato. http://www.cs.waikato.ac.nz/ml/weka/.
51. Gillespie DT (1977) Exact stochastic simulation of coupled chemical reactions. Journal of Physical Chemistry 81: 2340–2361.
52. Jeschke M, Ewald R, Uhrmacher AM (2011) Exploring the performance of spatial stochastic sim-ulation algorithms. Journal of Computational Physics 230: 2562–2574.
53. Banga J (2008) Optimization in computational systems biology. BMC Systems Biology 2: 47+.
54. Muggleton SH (2005) Machine learning for systems biology. In: Kramer S, Pfahringer B, edi-tors, Inductive Logic Programming, Springer Berlin Heidelberg, volume 3625 of Lecture Notes in Computer Science. pp. 416–423. URL http://dx.doi.org/10.1007/11536314_27. doi:10.1007/11536314_27
55. Asmussen S, Glynn PW (2007) Stochastic simulation. Springer.
56. Asmussen S, Glynn PW, Thorisson H (1992) Stationarity detection in the initial transient problem. ACM Transactions on Modeling and Computer Simulation (TOMACS) 2: 130–157.
57. Hoad K, Robinson S, Davies R (2008) Automating warm-up length estimation. In: Proceedings of the Winter Simulation Conference (WSC). pp. 532–540.
58. Pawlikowski K (1990) Steady-state simulation of queueing processes: A survey of problems and solutions. Computing Surveys 122: 123–170.
59. Kima M, Yoonb SH, Domanskib PA, Payneb WV (2007) Design of a steady-state detector for fault detection and diagnosis of a residential air conditioner. International Journal of Refrigeration 31: 791–792.
60. Box GEP, Pierce DA (1970) Distribution of residual correlations in autoregressive-integrated mov-ing average time series models. Journal of the American Statistical Association 65: 1509–1526.
61. Goodwin P (2010) The Holt-Winters approach to exponential smoothing: 50 years old and going strong. Foresight: The International Journal of Applied Forecasting.
62. Quinlan JR (1992) C4.5: programs for machine learning. Morgan Kaufmann, 1 edition.
63. Spratt SC (1998) An evaluation of contemporary heuristics for the startup problem. Master's thesis, University of Virginia.
64. White Jr KP, Cobb MJ, Spratt SC (2000) A comparison of five steady-state truncation heuristics for simulation. In: Proceedings of the Winter Simulation Conference (WSC). pp. 755–760.
65. Gafarian AV, Ancker CJ, Morisaku T (1976) The problem of the initial transient in digital computer simulation. In: Proceddings of the Winter Simulation Conference (WSC). pp. 49–51.
66. Gafarian AV, Ancker CJ, Morisaku T (1978) Evaluation of commonly used rules for detecting steady state. Computer Simulation 25: 511–529.
67. Wilson JR, Pritsker AAB (1978) Evaluation of startup policies in simulation experiments. Simu-lation 31: 79–89.
68. Milner R, Parrow J, Walker D (1992) A calculus of mobile processes, part i and ii. Information and Computation: 1–40, 41–77.
69. Maus C, Rybacki S, Uhrmacher AM (2011) Rule-based multi-level modeling of cell biological sys-tems. BMC Systems Biology 5: 77–91.
70. Jolliffe IT (2002) Principal Component Analysis. Springer Series in Statistics. Springer, 2nd edi-tion.
71. Rybacki S, Himmelspach J, Haack F, Uhrmacher AM (2011) WorMS - a framework to support workows in M&S. In: Proceedings of the Winter Simulation Conference (WSC). pp. 716–727.
